# New‐onset atrial fibrillation predicting for complicating cardiac adverse outcome in scrub typhus infection

**DOI:** 10.1002/clc.23276

**Published:** 2019-10-03

**Authors:** Suk‐Yong Jang, Ki‐Woon Kang, Jun Hyung Kim, Bongyoung Kim, Jung Yeon Chin, Sang Hyun Park, Yu Jeong Choi, Kyung Tae Jung, Seong‐Kyu Lee

**Affiliations:** ^1^ Department of Preventive Medicine Eulji University School of Medicine Daejeon Republic of Korea; ^2^ Department of Internal Medicine Eulji University School of Medicine Daejeon Republic of Korea; ^3^ Chungnam National University Daejeon Republic of Korea; ^4^ Department of Infection Hanyang University Hospital Seoul Republic of Korea

**Keywords:** Atrial fibrillation, Heart failure, Ischemic heart disease, Scrub typhus

## Abstract

**Background:**

Scrub typhus is a well‐known infectious disorder of the Asia‐Pacific region. However, adverse cardiac outcomes are an under‐recognized complication of scrub typhus infection, and new‐onset AF has been reported to be a prognostic factor in other, more common infectious diseases. The present study investigated whether new‐onset atrial fibrillation (AF) is significantly associated with 3‐month mortality and adverse cardiac complications in scrub typhus infection.

**Methods:**

We examined data from the National Health Information Database (NHID) which covers nearly the entire population of South Korea, from 2006 to 2016. In total, 233 473 patients diagnosed with scrub typhus infection were selected as study participants. New‐onset AF, acute heart failure (AHF), ischemic heart disease (IHD), and 3‐month mortality were analyzed using a generalized estimating equation model with a Poisson distribution.

**Results:**

Of these, 2402 patients (1%) were diagnosed with new‐onset AF (87.2% were over 60 years of age, 43.3% were male). Those with new‐onset AF were more likely to have underlying cardiovascular disease compared to those without new‐onset AF. After being adjusted for demographic factors and comorbidities, those with new‐onset AF had a higher incidence risk of concurrent AHF (4.1‐fold) and IHD (1.9‐fold) compared with those without new‐onset AF. In particular, the 3‐month mortality was also significantly associated with new‐onset AF (1.3‐fold), concurrent AHF (2.4‐fold), and IHD (13.7‐fold).

**Conclusions:**

New‐onset AF was significantly associated with 3‐month mortality and concurrent AHF and IHD. Therefore, new‐onset AF could be a poor prognostic factor for 3‐month mortality and cardiac complications in scrub typhus infection.

## 1 INTRODUCTION

Scrub typhus is a well‐known, seasonal infection caused by *Orientia tsutsugamushi*, which mainly confined to Southeastern Asia and the Western Pacific rim.^1^, ^2^ Recently, the geographical distribution of its endemic area has been widening with its overall mortality rate increasing. However, scrub‐typhus‐induced adverse cardiovascular complications remain remarkably under‐recognized.^3^ The majority of scrub typhus infections resolve with proper antibiotics and supportive treatment without any complications.^4^ However, with regards to scrub typhus infection complications,^5^, ^6^, ^7^ the overall mortality rate has been reported to range from overall 16% to 30%, which might be attributed to cardiovascular complications.^8^, ^9^ In particular, new‐onset atrial fibrillation (AF) has been reported as a poor prognostic factor in common infectious disorders,^10^, ^11^ which points to the need for investigating the association between scrub typhus infection and subsequent adverse cardiac events. New‐onset AF, acute heart failure (AHF), and ischemic heart disease (IHD) have been recognized as the major cardiac manifestations of public health adverse outcomes and the primary end points of infection‐induced cardiovascular outcomes.^11^, ^12^, ^13^ Therefore, we investigated whether new‐onset AF was significantly associated with 3‐month mortality and concurrent AHF and IHD in a nationwide cohort of scrub typhus infection.

## 2 METHODS

### 2.1 Data source

This study used data from the National Health Information Database (NHID) from 2006 to 2016. This is a public database on healthcare utilization, health screening, sociodemographic variables, and mortality for the entire population of South Korea (hereafter referred to as “Korea”), which was formed by the National Health Insurance Service (NHIS).^14^ Under universal medical coverage, all medical claims data are collected by the NHIS as the single insurer in Korea; therefore, all individuals included in the NHID were followed until 2017 unless there was a death or disqualification from National Health Insurance for an appropriate reason, such as emigration. The NHID includes an eligibility database, a national health screening database, a healthcare utilization database, a long‐term care insurance database, and a healthcare provider database.^14^ The healthcare utilization database is based on data collected during the processing of healthcare claims for services used and includes records of inpatient and outpatient usage (diagnosis, length of stay, treatment costs, services received) and prescription records (drug code, days prescribed, daily dosage).^14^ Access to the NHID can be obtained through the Health Insurance Data Service home page [http://nhiss.nhis.or.kr].

### 2.2 Sample size and collection

The population included in the NHID was over 49 million in 2006 and 51 million in 2016. From the NHID, during 2006 to 2016, a total of 240 329 patients with scrub typhus were selected using three criteria: (a) diagnosis code of ICD‐10 A753 (typhus fever due to *Rickettsia tsutsugamushi*), A752 (typhus fever due to *Rickettsia typhi*), or A759 (typhus fever, unspecified); (b) prescription of doxycycline or azithromycin for at least 3 days; and (c) 20 years of age or over at the time of diagnosis.^12^ In order to detect relevant cases, 2661 patients were excluded due to prior history of AF or acute myocarditis. Additionally, 4195 patients with missing values were excluded. Finally, a total of 233 473 patients with scrub typhus were selected as study participants. The index date (date of diagnosis) was defined as the date of first prescription.

### 2.3 Definition of new‐onset atrial fibrillation, acute heart failure, ischemic heart disease, and mortality

New‐onset AF was defined with a diagnosis code of ICD‐10 I48 (paroxysmal AF) within 30 days of the index date and no prior history of AF. New‐onset AHF was defined by a diagnosis code of ICD‐10 I40 (acute myocarditis), I30 (acute pericarditis), or I50 (heart failure) within 30 days of the index date. To exclude AHF induced by IHD, patients treated with coronary bypass graft surgery, primary coronary intervention, or thrombolytic agents (streptokinase, urokinase, tenecteplase) were further excluded. New‐onset IHD was defined as: (a) a diagnosis code of ICD‐10 I21 (acute myocardial infarction) or I20 (angina pectoris) within 30 days of the index date; and (b) treatment with coronary bypass graft surgery, primary coronary intervention, or thrombolytic agents (streptokinase, urokinase, tenecteplase). Three‐month all‐cause mortality was defined as death due to any cause within 90 days of the index date. Dates of deaths were obtained using each participant's unique, de‐identified number code, which is linked to mortality information from the Korean National Statistical Office.

### 2.4 Statistical analysis

To assess the association between new‐onset AF and the risk of AHF, IHD, and 30‐day mortality, a generalized estimating equation model with a Poisson distribution and logarithmic link function was used to estimate adjusted risk ratios (RRs) and 95% confidence intervals (CIs). Potential confounders were adjusted for using multivariable‐adjusted regression models. The participants' level of comorbidities were assessed using the diagnostic codes during the three years prior to the index date using the Quan's International Statistical Classification of Disease and Related Health Problems, 10th Revision (ICD‐10) coding algorithm of the Charlson Comorbidity Score (CCS).^15^ The presence of the disease categories AHF, IHD, stroke, chronic kidney disease, diabetes mellitus, hypertension, and malignancy were defined based on at least two outpatient visits or one inpatient admission with the corresponding primary or secondary diagnosis codes.

Statistical analyses were conducted using SAS software, version 9.4 (SAS Institute, Cary, North Carolina). A *P* value less than .05 was considered statistically significant.

## 3 RESULTS

### 3.1 Baseline characteristics

Most of the patients within the scrub typhus cohort were female residents (41.1%, male) in non‐metropolitan areas (75.0%) treated with doxycycline (93.7%). These patients and had low incidence of previous HF (1.2%), previous IHD (5.9%), chronic kidney disease (0.4%), diabetes (13.3%), or a CCS over three (5.1%).

### 3.2 Incidence of new‐onset AF

Of the 233 473‐scrub typhus infection records in the cohort, 2402 (1.0%) patients were diagnosed as having new‐onset AF during treatment for scrub typhus infection. Those with new‐onset AF tended to be over the age of 60 (87.2%) with significantly higher incidences of intensive care unit (ICU) hospitalization (7.7% vs 0.9%), previous HF (5.8% vs 1.2%), previous IHD (10.7% vs 5.9%), previous stroke (7.9% vs 3.5%), chronic kidney disease (1.0% vs 0.4%), diabetes (17.1% vs 13.2%), and hypertension (54.0% vs 34.0%) compared to those without new‐onset AF (Table 1).

**Table 1:** Baseline characteristics of scrub typhus patients with and without new‐onset atrial fibrillation

Variables	Total 233 473	With new‐onset AF 2402	Without AF 231 071	P
**Age**				<.0001
20‐49	48 519 (20.7%)	109 (4.5%)	48 410 (20.9%)	
50‐59	53 480 (22.9%)	199 (8.2%)	53 281 (23.0%)	
60‐69	60 676 (25.9%)	537 (22.3%)	60 139 (26.0%)	
70‐79	53 648 (22.9%)	972 (40.4%)	52 676 (22.8%)	
Over 80	17 150 (7.35%)	585 (24.3%)	16 565 (7.1%)	
**Sex**				0.0315
Male	96 071 (41.1%)	1040 (43.3%)	95 031 (41.1%)	
Female	137 402 (58.8%)	1362 (56.7%)	136 040 (58.8%)	
**Insurance**				
Medical aids	12 883 (5.5%)	207 (8.6%)	12 676 (5.4%)	
Health insurance				<.0001
1Q	38 844 (16.6%)	374 (15.5%)	38 470 (16.6%)	
2Q	42 517 (18.2%)	383 (15.9%)	42 134 (18.2%)	
3Q	58 837 (25.2%)	517 (21.5%)	58 320 (25.2%)	
4Q	80 392 (34.4%)	921 (38.3%)	79 471 (34.3%)	
**Residential area**				0.1446
Metropolitan	58 200 (24.9%)	568 (23.6%)	57 632 (24.9%)	
Non‐metropolitan	175 273 (75.0%)	1834 (76.3%)	173 439 (75.0%)	
**Institution**				<.0001
Outpatient care	96 057 (41.1%)	66 (2.7%)	95 991 (41.5%)	
**Admission**				
Less 300	84 847 (36.3%)	982 (40.8%)	83 865 (36.2%)	
300‐799	43 253 (18.5%)	1077 (44.8%)	42 176 (18.2%)	
Over 800	9316 (3.9%)	277 (11.5%)	9039 (3.9%)	
**Antibiotics**				<.0001
Doxycycline	218 791 (93.7%)	1932 (80.4%)	216 859 (93.8%)	
Azithromycin	8910 (3.8%)	227 (9.4%)	8683 (3.7%)	
Both	5772 (2.4%)	243 (10.1%)	5529 (2.3%)	
**ICU care**				<.0001
Yes	2342 (0.1%)	185 (7.7%)	2157 (0.9%)	
**Medical history**				
Past scrub typhus				<.0001
Yes	7300 (3.1%)	42 (1.7%)	7258 (3.1%)	
Congestive heart failure				<.0001
Yes	2973 (1.2%)	140 (5.8%)	2833 (1.2%)	
Ischemic heart disease				<.0001
Yes	13 995 (5.9%)	258 (10.7%)	13 737 (5.9%)	
Stroke				<.0001
Yes	8355 (3.5%)	190 (7.9%)	8165 (3.5%)	
Chronic kidney disease				<.0001
Yes	1121 (0.4%)	26 (1.0%)	1095 (0.4%)	
Diabetes mellitus				<.0001
Yes	31 072 (13.3%)	412 (17.1%)	30 660 (13.2%)	
Hypertension				<.0001
Yes	80 059 (34.2%)	1299 (54.0%)	78 760 (34.0%)	
Malignancy				0.7353
Yes	9979 (4.2%)	106 (4.4%)	9873 (4.2%)	
**Charlson Comorbidity Score**				<.0001
0	142 097 (60.8%)	1227 (51.0%)	140 870 (60.9%)	
1	57 174 (24.4%)	710 (29.5%)	56 464 (24.4%)	
2	22 228 (9.5%)	294 (12.2%)	21 934 (9.4%)	
Over 3	11 974 (5.1%)	171 (7.1%)	11 803 (5.1%)	
**Calendar year**				<.0001
2006	22 470 (9.6%)	221 (9.2%)	22 249 (9.6%)	
2007	19 413 (8.3%)	187 (7.7%)	19 226 (8.3%)	
2008	21 238 (9.0%)	199 (8.2%)	21 039 (9.1%)	
2009	22 726 (9.7%)	197 (8.2%)	22 529 (9.7%)	
2010	19 924 (8.5%)	228 (9.4%)	19 696 (8.5%)	
2011	18 435 (7.8%)	180 (7.4%)	18 255 (7.9%)	
2012	24 784 (10.6%)	230 (9.5%)	24 554 (10.6%)	
2013	24 547 (10.5%)	241 (10.0%)	24 306 (10.5%)	
2014	17 916 (7.6%)	243 (10.0%)	17 673 (7.6%)	
2015	20 397 (8.7%)	303 (12.6%)	20 094 (8.7%)	
2016	21 623 (9.2%)	173 (7.2%)	21 450 (9.2%)	

### 3.3 Incidence risk ratio for cardiovascular complication

**Figure 1:**
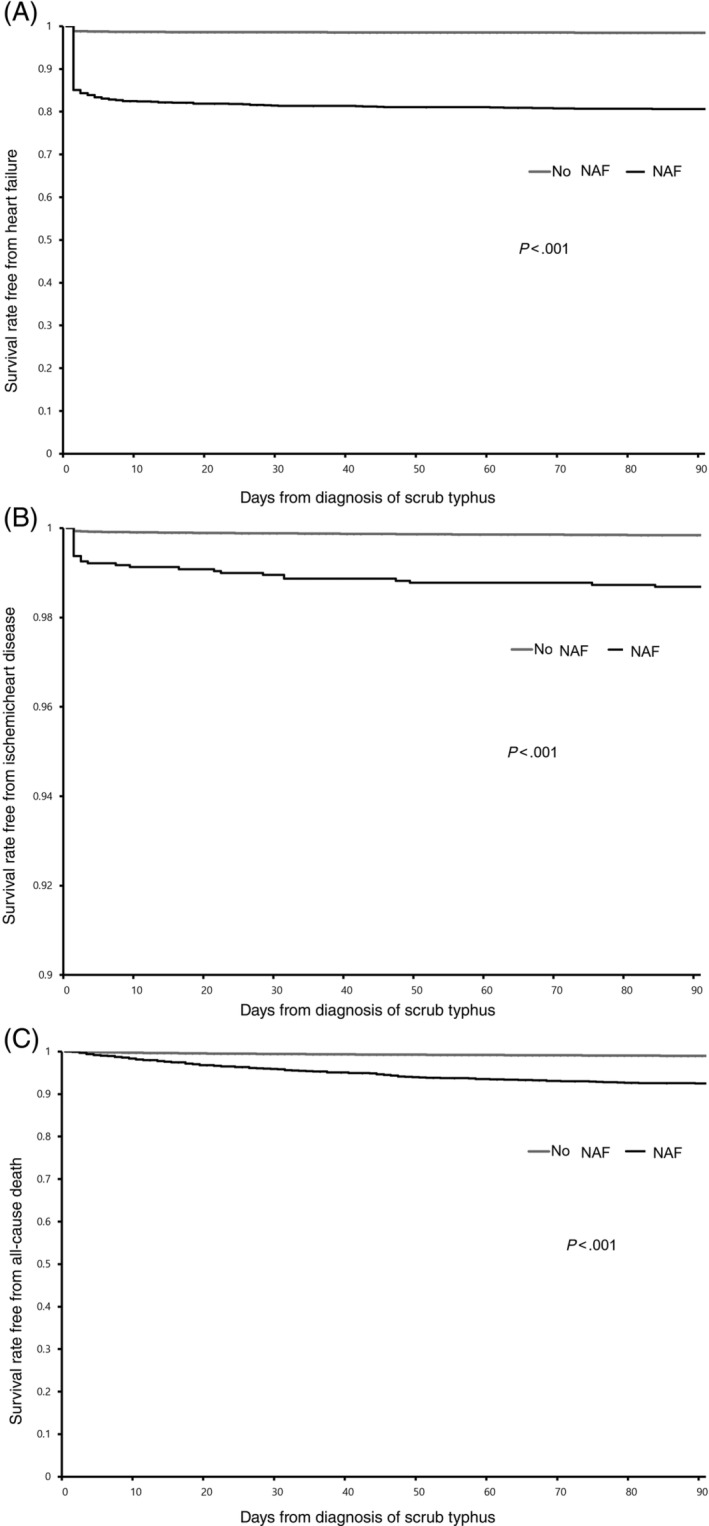
Comparison of survival rate free from, A, acute heart failure, B, ischemic heart disease, C, all‐cause mortality in patients diagnosed as scrub typhus between with and without new‐onset atrial fibrillation (NAF)

Those with new‐onset AF had an incidence risk ratio (IRR) of 4.1 for AHF within a few days of scrub typhus infection diagnosis compared with those without new‐onset AF (Figure 1A). Furthermore, patients over 50 years of age had an increased IRR of 2.0 to 5.4 for AHF. Patients admitted to the ICU or having a previous diagnosis of HF or IHD had an IRR for AHF of 2.4, 2.2, and 1.2, respectively, after being adjusted for demographic factors and comorbidities (Table 2).

**Table 2:** Incidence risk ratio for the occurrence of acute heart failure according to the development of new‐onset atrial fibrillation among scrub typhus patients

	Cumulative incidence			Crude model				Adjusted model			
Variables	Events	At risk	%	Relative risk	95% confidence interval		*P*	Adjust relative risk	95% confidence interval		*P*
**New‐onset AF**											
No	3273	231 071	1.42	1.00				1.00			
Yes	446	2402	18.57	**13.11**	11.98	14.35	<.0001	**4.12**	4.55	3.73	<.0001
**Age**											
20‐49	235	48 519	0.48	1.00				1.00			
50‐59	349	53 480	0.65	**1.35**	1.14	1.59	.0004	**1.27**	1.49	1.07	.0052
60‐69	711	60 676	1.17	**2.42**	2.09	2.80	<.0001	**2.02**	2.35	1.74	<.0001
70‐79	1450	53 648	2.70	**5.58**	4.86	6.40	<.0001	**3.59**	4.15	3.10	<.0001
Over 80	974	17 150	5.68	**11.73**	10.18	13.51	<.0001	**5.46**	6.36	4.69	<.0001
**Sex**											
Male	1335	96 071	1.39	**0.80**	0.75	0.86	<.0001	**0.94**	1.00	0.88	.0536
Female	2384	137 402	1.74	1.00				1.00			
**Insurance**											
Medical aids	352	12 883	2.73	1.00				1.00			
Health insurance											
1Q	630	38 844	1.62	**0.59**	0.52	0.68	<.0001	**0.89**	1.01	0.78	.0737
2Q	531	42 517	1.25	**0.46**	0.40	0.52	<.0001	**0.78**	0.89	0.68	.0002
3Q	803	58 837	1.36	**0.50**	0.44	0.57	<.0001	**0.80**	0.91	0.71	.0004
4Q	1403	80 392	1.75	**0.64**	0.57	0.72	<.0001	**0.78**	0.88	0.70	<.0001
**Residential area**											
Metropolitan	865	58 200	1.49	1.00				1.00			
Non‐metropolitan	2854	175 273	1.63	**0.91**	0.85	0.98	.0178	**0.97**	1.05	0.90	.5052
**Institution**											
Outpatient care	115	96 057	0.12	1.00				1.00			
**Antibiotics**											
Doxycycline	3016	218 791	1.38	1.00				1.00			
Azithromycin	382	8910	4.29	**3.11**	2.80	3.45	<.0001	**1.59**	1.77	1.43	<.0001
Both	321	5772	5.56	**4.03**	3.61	4.51	<.0001	**1.52**	1.70	1.35	<.0001
**ICU care**											
No	3510	231 131	1.52	1.00				1.00			
Yes	209	2342	8.92	**5.88**	5.14	6.72	<.0001	**2.46**	2.84	2.13	<.0001
**Medical history**											
Past scrub typhus											
No	3628	226 173	1.60	1.00				1.00			
Yes	91	7300	1.25	**0.78**	0.63	0.96	.0169	**0.81**	0.99	0.66	.0417
Congestive heart failure											
No	3492	230 500	1.52	1.00				1.00			
Yes	227	2973	7.64	**5.04**	4.43	5.74	<.0001	**2.20**	2.53	1.92	<.0001
Ischemic heart disease											
No	3251	219 478	1.48	1.00				1.00			
Yes	468	13 995	3.34	**2.26**	2.05	2.48	<.0001	**1.28**	1.42	1.16	<.0001
Stroke											
No	3439	225 118	1.53	1.00				1.00			
Yes	280	8355	3.35	**2.19**	1.95	2.47	<.0001	**1.09**	1.23	0.96	.1742
Chronic kidney disease											
No	3673	232 352	1.58	1.00				1.00			
Yes	46	1121	4.10	**2.60**	1.95	3.45	<.0001	**1.05**	1.42	0.78	.7467
Diabetes mellitus											
No	2978	202 401	1.47	1.00				1.00			
Yes	741	31 072	2.38	**1.62**	1.50	1.76	<.0001	**1.02**	1.10	0.94	.6843
Hypertention											
No	1713	153 414	1.12	1.00				1.00			
Yes	2006	80 059	2.51	**2.24**	2.11	2.39	<.0001	**1.16**	1.25	1.08	<.0001
Malignancy											
No	3539	223 494	1.58	1.00				1.00			
Yes	180	9979	1.80	**1.14**	0.98	1.32	.0852	**0.86**	1.02	0.72	.0767
**Charlson Comorbidity Score**											
0	1838	142 097	1.29	1.00				1.00			
1	1036	57 174	1.81	**1.40**	1.30	1.51	<.0001	**0.95**	1.02	0.88	.1554
2	544	22 228	2.45	**1.89**	1.72	2.08	<.0001	**1.09**	1.21	0.99	.0896
Over 3	301	11 974	2.51	**1.94**	1.72	2.19	<.0001	**0.96**	1.11	0.83	.5876

Those with new‐onset AF had an IRR of 1.9 for IHD compared with those without new‐onset AF (Figure 1B). Patients over the age of 50 also had an increased IRR for IHD of 2.9 to 14.6. Those admitted to the ICU, or having a past scrub typhus infection, HF or IHD had an IRR for IHD of 5.6, 2.0, 1.5, and 2.0, respectively, after being adjusted for demographic factors and comorbidities (Table 3).

**Table 3:** Incidence risk ratio for the occurrence of ischemic heart disease according to the development of new‐onset atrial fibrillation among scrub typhus patients

	Cumulative incidence			Crude model				Adjusted model			
Variables	Events	At risk	%	Relative risk	95% confidence interval		*P*	Adjust relative risk	95% confidence interval		*P*
**New‐onset AF**								1.00			
No	278	231 071	0.12	1.00							
Yes	27	2402	1.12	**9.34**	6.31	13.84	<.0001	**1.95**	3.04	1.25	.0032
**Age**											
20‐49	8	48 519	0.02	1.00				1.00			
50‐59	26	53 480	0.05	**2.95**	1.34	6.51	.0075	**2.99**	6.64	1.35	.0071
60‐69	65	60 676	0.11	**6.50**	3.12	13.54	<.0001	**5.45**	11.51	2.58	<.0001
70‐79	128	53 648	0.24	**14.47**	7.08	29.56	<.0001	**9.60**	19.94	4.62	<.0001
Over 80	78	17 150	0.45	**27.58**	13.33	57.09	<.0001	**14.65**	31.00	6.92	<.0001
**Sex**											
Male	164	96 071	0.17	**1.66**	1.33	2.08	<.0001	**1.96**	2.46	1.56	<.0001
Female	141	137 402	0.10	1.00				1.00			
**Insurance**											
Medical aids	42	12 883	0.33	1.00				1.00			
Health insurance											
1Q	47	38 844	0.12	**0.37**	0.24	0.56	<.0001	**0.61**	0.92	0.40	.0197
2Q	44	42 517	0.10	**0.32**	0.21	0.48	<.0001	**0.55**	0.86	0.36	.0078
3Q	61	58 837	0.10	**0.32**	0.21	0.47	<.0001	**0.51**	0.77	0.34	.0013
4Q	111	80 392	0.14	**0.42**	0.30	0.60	<.0001	**0.52**	0.74	0.36	.0003
**Residential area**											
Metropolitan	81	58 200	0.14	1.00				1.00			
Non‐metropolitan	224	175 273	0.13	**1.09**	0.84	1.40	.5105	**1.16**	1.50	0.90	.2538
**Institution**											
Outpatient care	19	96 057	0.02	1.00				1.00			
**Antibiotics**											
Doxycycline	221	218 791	0.10	1.00				1.00			
Azithromycin	35	8910	0.39	**3.89**	2.72	5.55	<.0001	**1.97**	2.86	1.35	.0004
Both	49	5772	0.85	**8.40**	6.17	11.44	<.0001	**3.45**	4.79	2.48	<.0001
**ICU care**											
No	259	231 131	0.11	1.00				1.00			
Yes	46	2342	1.96	**17.53**	12.84	23.92	<.0001	**5.63**	7.99	3.97	<.0001
**Medical history**											
Past scrub typhus											
No	289	226 173	0.13	1.00				1.00			
Yes	16	7300	0.22	**1.72**	1.04	2.84	.0354	**2.08**	3.44	1.25	.0046
Congestive heart failure											
No	290	230 500	0.13	1.00				1.00			
Yes	15	2973	0.50	**4.01**	2.39	6.73	<.0001	**1.50**	2.54	0.88	.1342
Ischemic heart disease											
No	247	219 478	0.11	1.00				1.00			
Yes	58	13 995	0.41	**3.68**	2.77	4.90	<.0001	**2.00**	2.71	1.47	<.0001
Stroke											
No	281	225 118	0.12	1.00				1.00			
Yes	24	8355	0.29	**2.30**	1.52	3.49	<.0001	**0.94**	1.45	0.61	.7672
Chronic kidney disease											
No	293	232 352	0.13	1.00				1.00			
Yes	12	1121	1.07	**8.49**	4.78	15.07	<.0001	**2.67**	5.16	1.38	.0035
Diabetes mellitus											
No	232	202 401	0.11	1.00				1.00			
Yes	73	31 072	0.23	**2.05**	1.58	2.67	<.0001	**1.22**	1.62	0.92	.1583
Hypertension											
No	130	153 414	0.08	1.00				1.00			
Yes	175	80 059	0.22	**2.58**	2.06	3.24	<.0001	**1.25**	1.62	0.96	.0929
Malignancy											
No	287	223 494	0.13	1.00				1.00			
Yes	18	9979	0.18	**1.40**	0.87	2.26	.1616	**0.93**	1.61	0.54	.8009
**Charlson Comorbidity Score**											
0	140	142 097	0.10	1.00				1.00			
1	89	57 174	0.16	**1.58**	1.21	2.06	.0007	**1.02**	1.34	0.77	.8893
2	43	22 228	0.19	**1.96**	1.40	2.76	.0001	**0.98**	1.41	0.69	.9271
Over 3	33	11 974	0.28	**2.80**	1.92	4.09	<.0001	**0.97**	1.61	0.58	.8956

### 3.4 Cardiovascular complications and mortality

New‐onset AF, AHF and IHD had an IRR for 3‐month mortality of 1.3, 2.4, and 13.7, respectively, after controlling for demographic factors and comorbidities. Increased age over 50 years also had an increased IRR of 1.8 to 10.2 for 3‐month mortality, and those admitted to the ICU had an IRR for 3‐month mortality of 4.5. Interestingly, those with better economic or health status also showed a lower IRR for mortality than those with poorer economic or health status (Table 4, Figure 1C).

**Table 4:** Incidence risk ratio of demographic characteristics and comorbidities for mortality in scrub typhus patients

	Cumulative incidence			Crude model				Adjusted model			
Variables	Events	At risk	%	Relative risk	95% confidence interval		*P*	Adjust relative risk	95% confidence interval		*P*
**New‐onset AF**											
No	1347	231 071	0.58	1.00				1.00			
Yes	105	2402	4.37	**7.50**	6.17	9.11	<.0001	**1.34**	1.07	1.68	.0106
**Acute heart failure**											
No	1243	229 754	0.54	1.00				1.00			
Yes	209	3719	5.62	**10.39**	9.00	11.98	<.0001	**2.41**	2.04	2.84	<.0001
**Ischemic heart disease**											
No	1292	233 168	0.55	1.00				1.00			
Yes	160	305	52.46	**94.67**	83.98	106.73	<.0001	**13.72**	11.03	17.07	<.0001
**Age**											
20‐49	62	48 519	0.13	1.00				1.00			
50‐59	121	53 480	0.23	**1.77**	1.30	2.40	.0003	**1.80**	1.33	2.44	.0001
60‐69	217	60 676	0.36	**2.80**	2.11	3.71	<.0001	**2.39**	1.79	3.18	<.0001
70‐79	569	53 648	1.06	**8.30**	6.39	10.78	<.0001	**5.33**	4.06	7.02	<.0001
Over 80	483	17 150	2.82	**22.04**	16.93	28.69	<.0001	**10.29**	7.76	13.65	<.0001
**Sex**											
Male	751	96 071	0.78	**1.53**	1.38	1.70	<.0001	**1.68**	1.51	1.87	<.0001
Female	701	137 402	0.51	1.00				1.00			
**Insurance**											
Medical aids	181	12 883	1.41	1.00				1.00			
Health insurance											
1Q	233	38 844	0.60	**0.43**	0.35	0.52	<.0001	**0.77**	0.63	0.94	.0118
2Q	215	42 517	0.51	**0.36**	0.30	0.44	<.0001	**0.73**	0.60	0.90	.0033
3Q	308	58 837	0.52	**0.37**	0.31	0.45	<.0001	**0.70**	0.58	0.85	.0002
4Q	515	80 392	0.64	**0.46**	0.39	0.54	<.0001	**0.63**	0.52	0.75	<.0001
**Residential area**											
Metropolitan	302	58 200	0.52	1.00				1.00			
Non‐metropolitan	1150	175 273	0.66	**0.79**	0.70	0.90	.0003	**0.86**	0.75	0.98	.0292
**Institution**											
Outpatient care	72	96 057	0.08	1.00				1.00			
Admission											
**Antibiotics**											
Doxycycline	952	218 791	0.44	1.00				1.00			
Azithromycin	310	8910	3.48	**8.00**	7.05	9.07	<.0001	**3.43**	2.97	3.96	<.0001
Both	190	5772	3.29	**7.57**	6.49	8.82	<.0001	**2.45**	2.07	2.90	<.0001
**ICU care**											
No	1241	231 131	0.54	1.00				1.00			
Yes	211	2342	9.01	**16.78**	14.59	19.30	<.0001	**4.51**	3.77	5.39	<.0001
**Medical history**											
Past scrub typhus											
No	1388	226 173	0.61	1.00				1.00			
Yes	64	7300	0.88	**1.43**	1.11	1.83	.0051	**1.19**	0.91	1.55	.213
Congestive heart failure											
No	1385	230 500	0.60	1.00				1.00			
Yes	67	2973	2.25	**3.75**	2.94	4.78	<.0001	**1.45**	1.10	1.91	.0083
Ischemic heart disease											
No	1294	219 478	0.59	1.00				1.00			
Yes	158	13 995	1.13	**1.91**	1.62	2.26	<.0001	**1.01**	0.85	1.20	.9023
Stroke											
No	1316	225 118	0.58	1.00				1.00			
Yes	136	8355	1.63	**2.78**	2.34	3.32	<.0001	**1.10**	0.91	1.32	.3361
Chronic kidney disease											
No	1412	232 352	0.61	1.00				1.00			
Yes	40	1121	3.57	**5.87**	4.31	8.00	<.0001	**1.18**	0.81	1.72	.3797
Diabetes mellitus											
No	1120	202 401	0.55	1.00				1.00			
Yes	332	31 072	1.07	**1.93**	1.71	2.18	<.0001	**1.29**	1.13	1.47	.0001
Hypertension											
No	704	153 414	0.46	1.00				1.00			
Yes	748	80 059	0.93	**2.04**	1.84	2.26	<.0001	**0.94**	0.84	1.06	.3192
Malignancy											
No	1327	223 494	0.59	1.00				1.00			
Yes	125	9979	1.25	**2.11**	1.76	2.53	<.0001	**0.99**	0.77	1.26	.9164
**Charlson Comorbidity Score**											
0	565	142 097	0.40	1.00				1.00			
1	443	57 174	0.77	**1.95**	1.72	2.21	<.0001	**1.32**	1.16	1.49	<.0001
2	227	22 228	1.02	**2.57**	2.20	2.99	<.0001	**1.31**	1.11	1.55	.0015
Over 3	217	11 974	1.81	**4.56**	3.90	5.32	<.0001	**1.76**	1.43	2.17	<.0001

## 4 DISCUSSION

In this nationwide scrub typhus infection cohort, patients with new‐onset AF were more likely to be hospitalized in the ICU and had higher 3‐month mortality rates. In particular, new‐onset AF was significantly associated with concurrent AHF or IHD during treatment for scrub typhus infection. Unlike the adverse cardiac complications occurring as a result of common infections,^16^, ^17^ evidence to date has been unclear concerning an association between scrub typhus infection and adverse cardiac outcomes.^11^ The present study is the first to demonstrate that new‐onset AF was significantly associated with 3‐month mortality and adverse cardiac complications in scrub typhus infection.

Occurrence of new‐onset AF has been known to be associated with infection which may be triggered by acute inflammatory condition.^18^ In critically‐ill patients with common infectious diseases, new‐onset AF has been reported to be significantly associated with all‐cause mortality in the ICU.^10^, ^19^ The FROG‐ICU trial demonstrated that new‐onset AF occurred in 19% of all patients in the ICU and had an incidence risk of 2.2‐fold for 1‐year mortality compared to those without new‐onset AF.^20^ The present study demonstrates that new‐onset AF occurred in the 7.7% of all patients in the ICU and had an incidence risk of 4.5‐fold for 3‐month mortality compared to those without new‐onset AF. It is noteworthy that new‐onset AF in scrub typhus infection developed less frequently, but had a higher risk of mortality than in the other infectious diseases. The reason for the higher mortality and adverse cardiac complications could be explained by the unique pathophysiology of scrub typhus infection,^17^ which initiates at the site of skin inoculation, evolves into regional lymphadenopathy and spreads to vasculitis with subsequent target organ damage.^21^ Subsequently, induced myocardial inflammation could develop electrical, functional, and structural remodeling during the pathogenesis of new‐onset AF and AHF.^22^, ^23^, ^24^, ^25^, ^26^ The present study also demonstrates that AHF concurrent with new‐onset AF could develop within only a few days of the index diagnosis of scrub typhus infection (Figure 1A). In addition, new‐onset AF was also associated with a greater risk for developing IHD in the critically‐ill status including complicating scrub typhus infection.^11^, ^27^, ^28^ Coronary vasculitis also might induce direct endothelial dysfunction and vascular injury causing atherosclerotic plaque growth or rupture during the pathogenesis of IHD.^29^, ^30^ In particular, ECG or rhythm surveillance for cardiac complications could be a necessary monitoring of scrub typhus infection because of the risk of developing atrial or ventricular arrhythmia and changes in the ST segment of ECG as a result of active inflammation in the myocardium.^11^ Available ECG‐based new‐onset AF or ST segment change could be more readily evaluated^31^ than time and cost‐consuming echocardiogram‐based AHF or angiogram‐based IHD^32^ under the care of non‐cardiologic department. ECG or rhythm‐based surveillance for the development of cardiac complications is crucial for preventing scrub typhus infection from developing life‐threatening outcomes.^33^ This could provide an additional method for reducing adverse cardiac complications in scrub typhus infection.

### 4.1 Limitations

There are several limitations to the present study. First, the national cohort data does not include lifestyle information, such as alcohol intake, smoking habits, body mass index or family history, all of which are potential confounding factors in this study. Second, old age, hypertension, diabetes and previous HF are well‐known comorbidities strongly correlated with new‐onset AF. Therefore, we adjusted for these comorbidities to minimize the influence of AHF or IHD on 3‐month mortality. Third, living in a metropolitan area or being treated with azithromycin for a refractory or complicated type of scrub typhus infection also might induce treatment bias. Fourth, the cohort data were selected according to ICD codes, which may potentially have misclassification bias. Fifth, there was no control group of patients without scrub typhus infection. Therefore, our results might not be fully generalizable, and a prospective, randomized controlled trial should be conducted to overcome these limitations.

## 5 CONCLUSION

New‐onset AF was significantly associated with 3‐month mortality and concurrent cardiac adverse outcomes. Therefore, new‐onset AF may be a poor prognostic factor for 3‐month mortality and adverse cardiac complications in scrub typhus infection. Further investigation is warranted to prospectively validate these results.

## CONFLICT OF INTEREST

The authors declare no potential conflict of interests.

## AUTHOR CONTRIBUTIONS

K.W. K. contributed to the study design, interpretation of the analyzed data, and revised final manuscript. S.Y. J. collected and analyzed the data. J. H. K., B. K., J. Y. C., S. H. P., Y. J. C., K.T. J., and S.K. L. interpreted the data and drafted the manuscript.

## ETHICS STATEMENT

This study was approved by the Institutional Review Board of Eulji University (EMC 2017‐10‐006) and adhered to the principles of the Declaration of Helsinki.
